# Ruptured Distal Medial Lenticulostriate Artery Aneurysm Treated With Transcortical-Transventricular Approach

**DOI:** 10.7759/cureus.16186

**Published:** 2021-07-05

**Authors:** Michael Young, Peter Schaible, Khaled Asi, Keith Schaible

**Affiliations:** 1 Neurological Surgery, Advocate Health Care, Oak Lawn, USA; 2 Neurological Surgery, Advocate Christ Medical Center, Oak Lawn, USA; 3 Endovascular Surgical Neuroradiology, Advocate Christ Medical Center, Oak Lawn, USA

**Keywords:** medial lenticulostriate artery aneurysm, transcortical transventricular, arteriovenous malformation, open surgical approach, endovascular, ruptured aneurysm, caudate nucleus hemorrhage, intraventricular hemorrhage

## Abstract

We report a case of a 48-year-old female who presented with abulia and headaches. Head CT (HCT) demonstrated a left caudate intracerebral hemorrhage (ICH) with extension into the left lateral ventricle. Diagnostic cerebral angiogram showed a left distal medial lenticulostriate artery (MLSA) aneurysm with remote left parietal Spetzler-Martin grade 3 arteriovenous malformation (AVM). The patient underwent an endoscope-assisted transcortical-transventricular approach to the distal MLSA aneurysm with complete excision and evacuation of the intraventricular hemorrhage (IVH) postoperatively; the patient had no further neurologic deficits and recovered well from her initial hemorrhage.

Ruptured MLSA aneurysms are a very rare cause of spontaneous ICH. Urgent treatment of these aneurysms is needed to prevent further rebleeding. We present a novel operative technique utilizing an endoscope-assisted transcortical-transventricular approach to a ruptured distal MLSA aneurysm with successful excision of the aneurysm.

## Introduction

Medial lenticulostriate artery (MLSA) aneurysms are a rare cerebrovascular pathology. Treatment options include observation, endovascular therapy, or open surgery. Ruptured MLSA aneurysms necessitate expedited treatment due to the fragile nature of these aneurysms and the risk of rebleeding. There are few reported cases in the literature about the management of these rare aneurysms. We present a case of a transcortical-transventricular approach to a ruptured MLSA aneurysm with successful obliteration and a good neurological outcome.

## Case presentation

**Figure 1:**
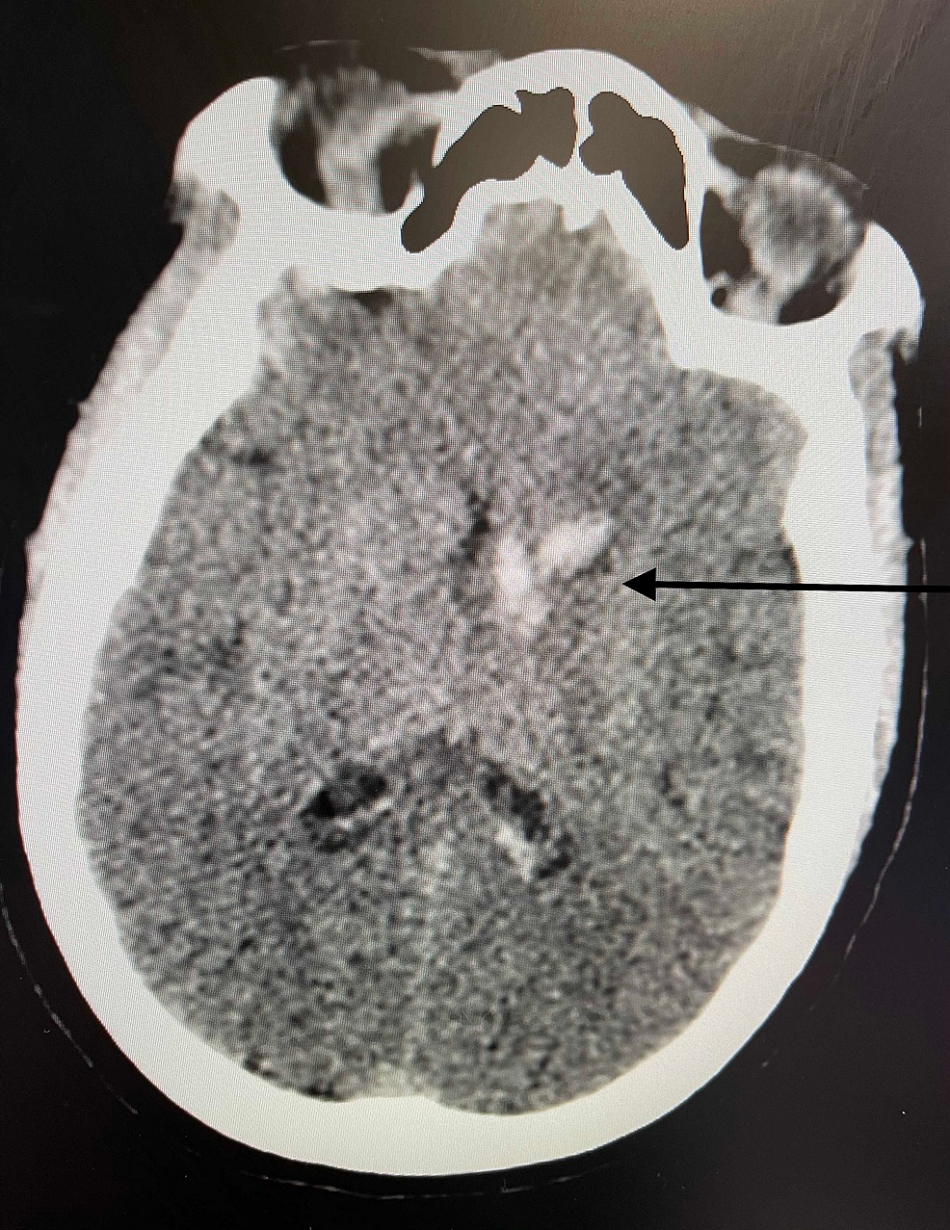
Initial head CT demonstrating left caudate nucleus intracerebral hemorrhage with left lateral ventricle intraventricular hemorrhage (arrow) CT: computed tomography

A 48-year-old female with a history of hypertension presented with acute onset of lethargy after a four-day history of headaches, and abulia. On presentation, she had a Glasgow Coma Scale (GCS) score of 15 with non-focal neurologic examination. Non-contrast head CT (NCHCT) demonstrated a left caudate intracerebral hemorrhage (ICH) with intraventricular hemorrhage (IVH) (Figure 1).

**Figure 2:**
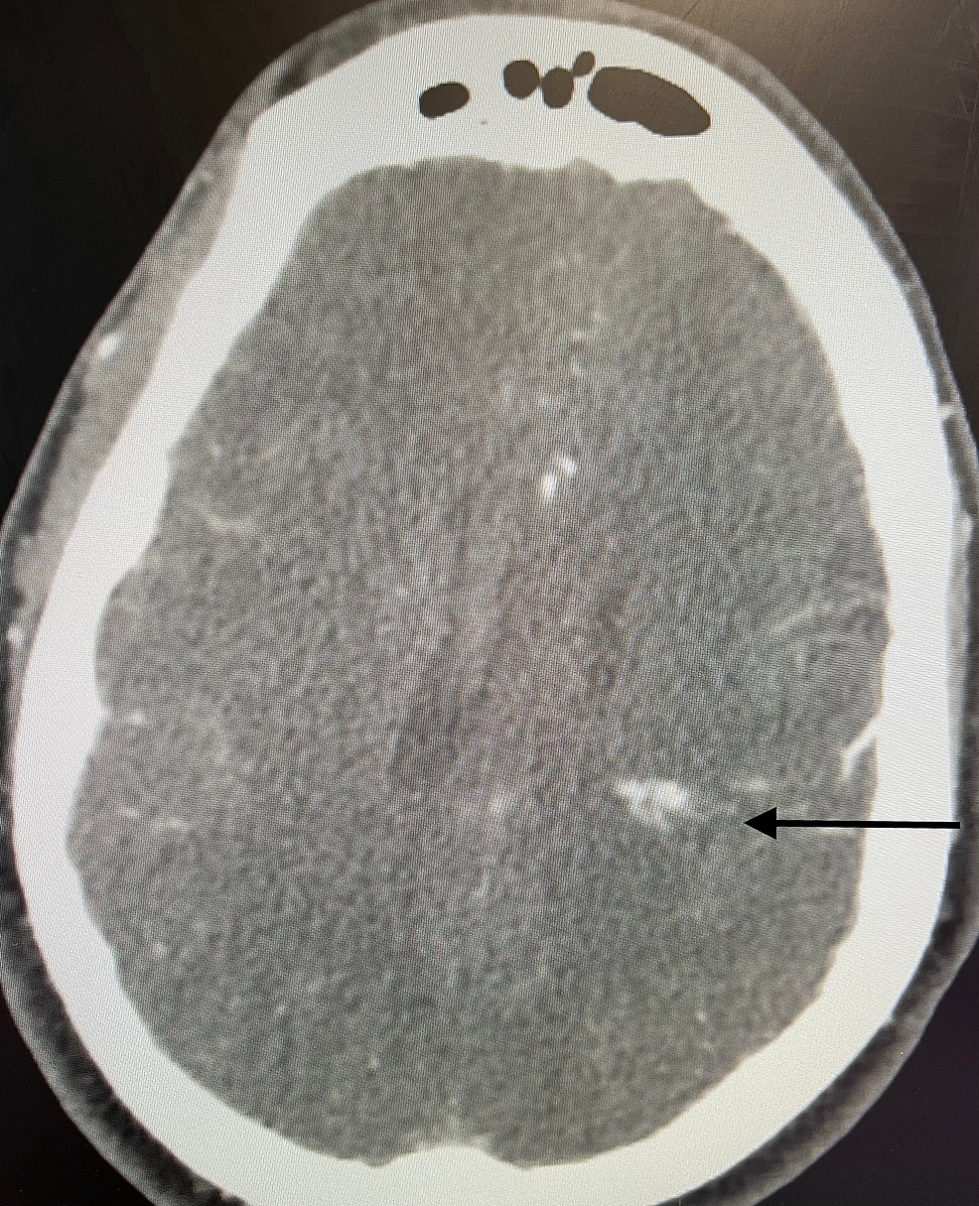
CTA demonstrating left parietal arteriovenous malformation (arrow) CTA: computed tomography angiography

CT angiography (CTA) failed to demonstrate a vascular lesion in proximity to the ICH and IVH but did show a left parietal arteriovenous malformation (AVM) remote to the caudate hemorrhage and IVH (Figure 2).

**Figure 3:**
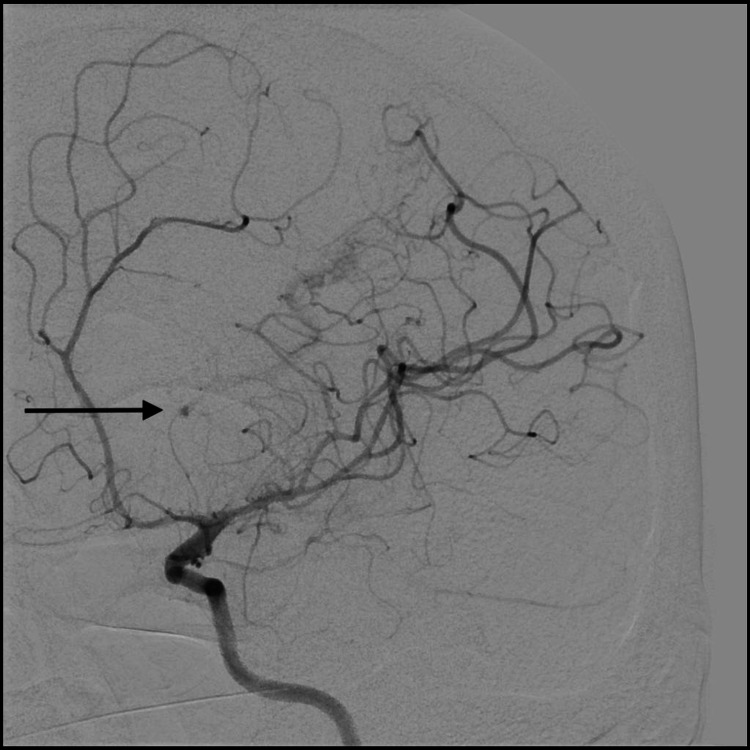
AP cerebral angiogram demonstrating left distal MLSA aneurysm (arrow) AP: anteroposterior; MLSA: medial lenticulostriate artery

**Figure 4:**
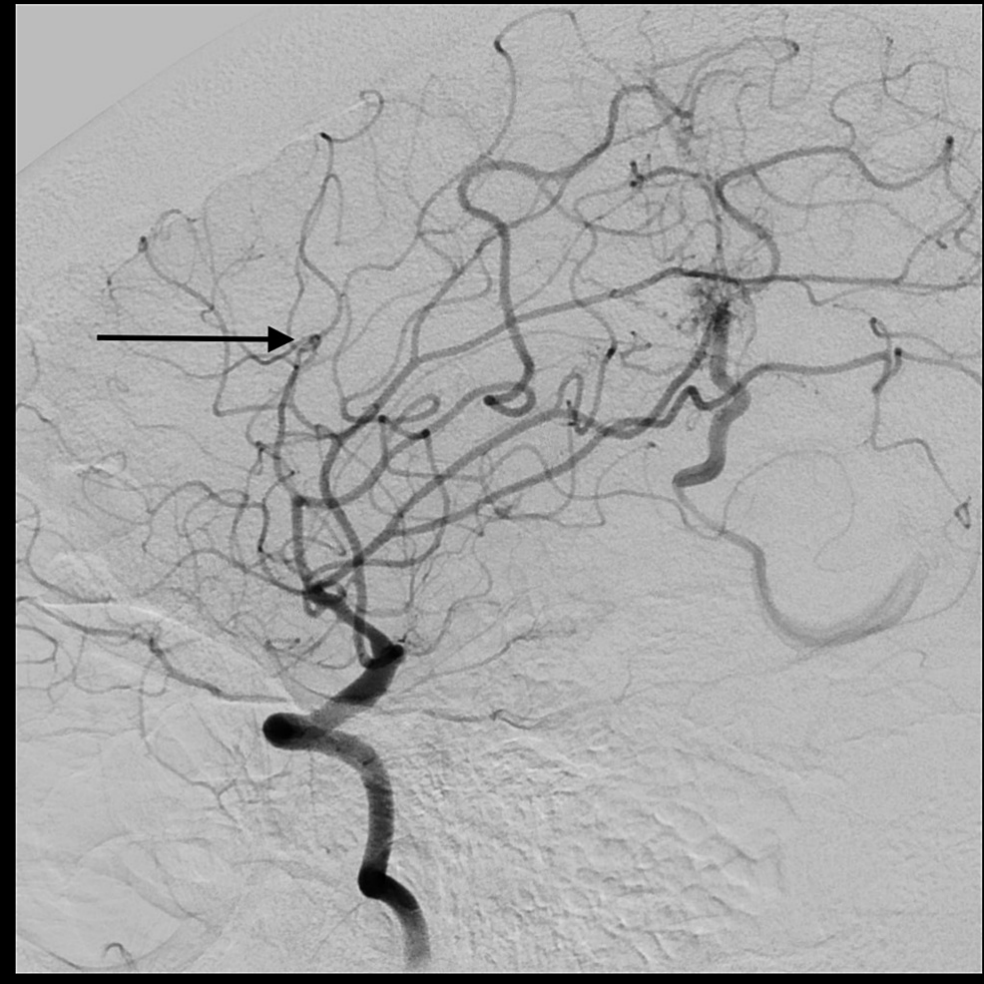
Lateral cerebral angiogram demonstrating left distal MLSA aneurysm (arrow) MLSA: medial lenticulostriate artery

The patient then underwent a diagnostic cerebral angiogram, which demonstrated a left MLSA aneurysm, and it was deemed to be the source of the ICH and IVH (Figures 3, 4).

Furthermore, the cerebral angiogram redemonstrated a left parietal Spetzler-Martin grade 3 AVM with arterial feeders from the angular branch of the left middle cerebral artery (MCA) and venous drainage into the internal cerebral veins. The aneurysm was remote from the AVM and hence clearly not a feeding artery aneurysm. Due to the very small caliber of the MLSA, endovascular access was deemed high risk for vessel rupture, and therefore surgical treatment was indicated.

**Figure 5:**
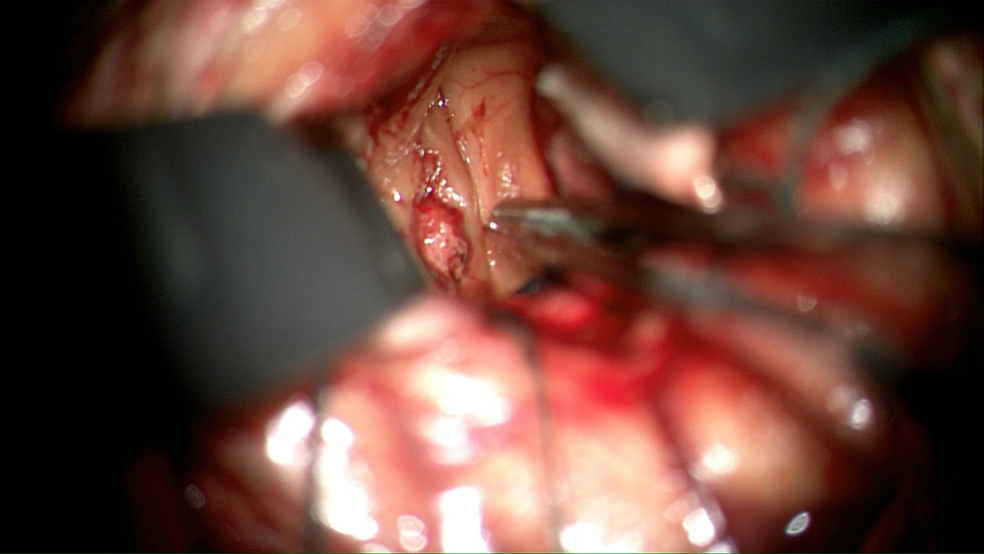
MLSA aneurysm approached via transcortical-transventricular approach MLSA: medial lenticulostriate artery

**Figure 6:**
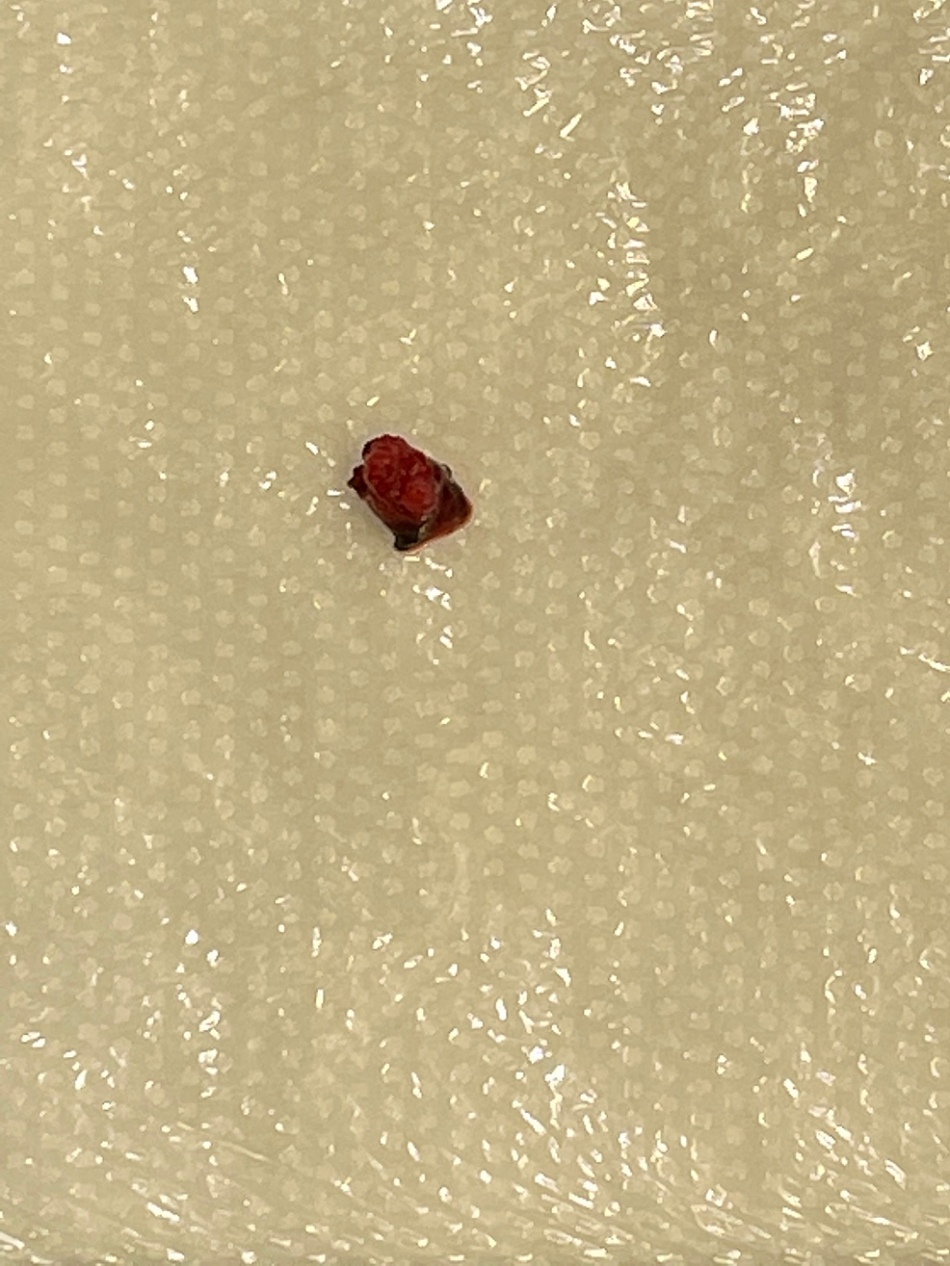
Excised MLSA aneurysm MLSA: medial lenticulostriate artery

The patient went to the operating room for a transcortical-transventricular approach to the MLSA aneurysm. Using image guidance, a small left frontal cortical opening was performed, anterior to the supplementary motor and primary motor cortices. An endoscopic sheath and endoscope were advanced into the left lateral ventricle with egress of cerebrospinal fluid (CSF). Due to poor visualization with the endoscope, the endoscope was removed, and the operative microscope was brought into the field. The endoscopic sheath provided a minimally invasive corticotomy to the left lateral IVH and caudate ICH. Using the microscope, the caudate ICH and left lateral ventricle IVH were evacuated. Once again, using image guidance, the MLSA aneurysm was identified within the caudate ICH evacuation cavity, and a small mini aneurysm clip was placed on the distal MLSA in attempts at proximal control. Upon dissection of the MLSA aneurysm, there was a small amount of hemorrhage, which was quickly controlled via bipolar cautery. The MLSA aneurysm was then completely excised and an external ventricular drain (EVD) was placed within the left lateral ventricle (Figures 5, 6; Video 1).

**Video 1:** Operative video demonstrating a transcortical-transventricular approach to MLSA aneurysm with successful excision MLSA: medial lenticulostriate artery

**Figure 7:**
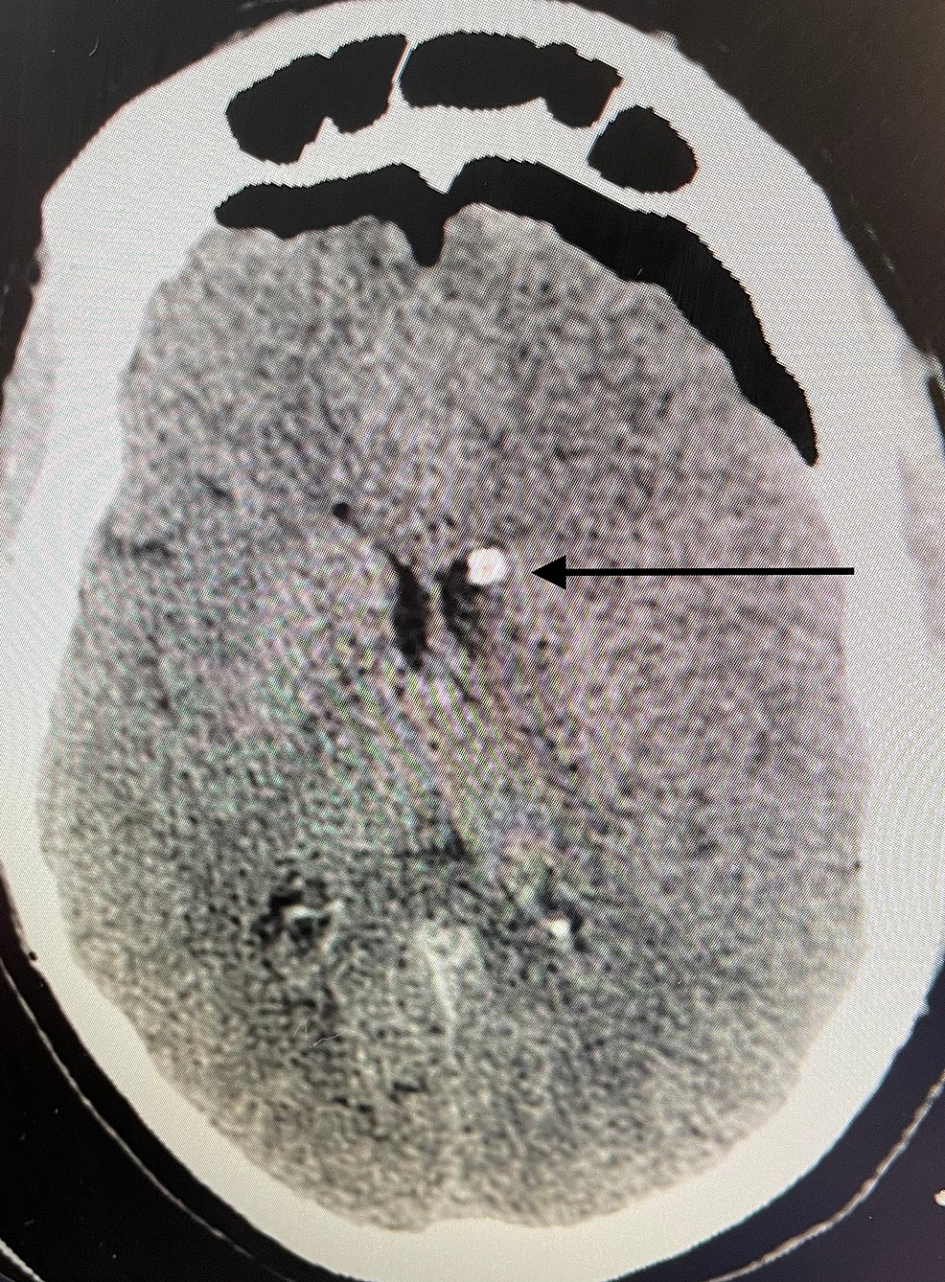
Postoperative head CT demonstrating complete resolution of left caudate nucleus intracerebral hemorrhage and left lateral ventricle intraventricular hemorrhage (arrow) CT: computed tomography

**Figure 8:**
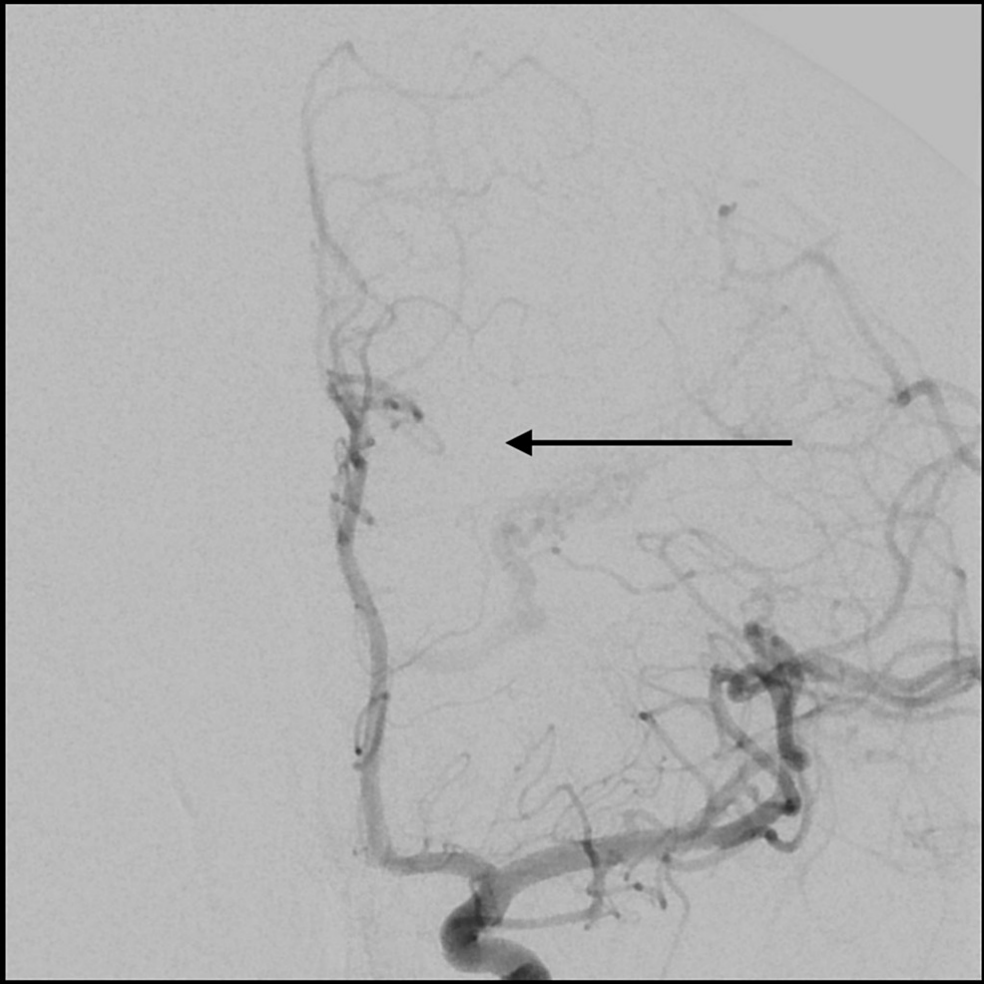
Anteroposterior postoperative cerebral angiogram demonstrating no further filling of the left MLSA aneurysm (arrow) MLSA: medial lenticulostriate artery

**Figure 9:**
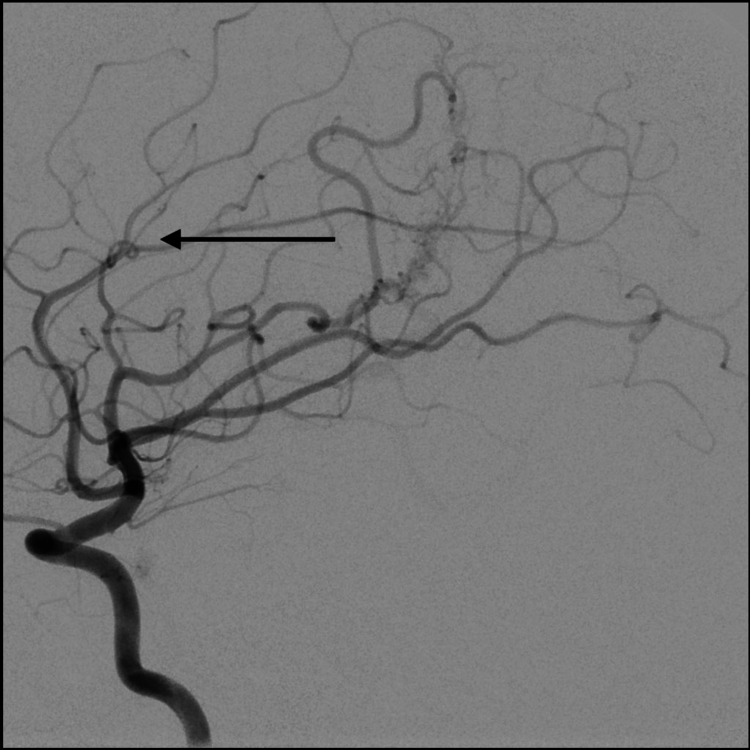
Lateral postoperative cerebral angiogram demonstrating no further filling of the left MLSA aneurysm (arrow) MLSA: medial lenticulostriate artery

Postoperative HCT showed the resolution of the caudate hemorrhage and left lateral ventricle IVH and diagnostic cerebral angiogram showed no further filling of the left distal MLSA aneurysm (Figures 7, 8, 9).

The EVD was then weaned off over several days and was able to be removed without the need for a ventriculoperitoneal shunt. The patient recovered well from her initial sequelae of hemorrhage with improvement in lethargy and no further neurologic deficits.

## Discussion

Basal ganglia hemorrhages are frequently seen in clinical practice. The etiology of such hemorrhages is most often hypertensive in nature. Other etiologies include intraventricular tumor, venous thrombosis, coagulopathy, moyamoya disease, or trauma [1]. However, as seen in our case, such a hemorrhage may harbor an underlying vascular lesion. Patients of younger age presenting with mild or minimal history of hypertension or hemorrhages associated with extension into the ventricular or subarachnoid space should prompt further diagnostic investigation with CTA or catheter-based cerebral angiography [2].

The lenticulostriate arteries are classified into the lateral and medial lenticulostriate arteries (LSAs). The lateral LSAs are classified as internal and external, and the medial LSA is classified as medial and the Heubner artery with the medial portion originating from the A1 portion of the anterior cerebral artery [3]. The MLSAs mainly supply the area of the inferior medial caudate nucleus, putamen, anterior paleostriatum, and genu and posterior limb of the internal capsule [4,5]. In contrast, the lateral LSAs supply the putamen, the adjacent part of the paleostriatum, the total rostrocaudal length of the upper part of the internal capsule, and the body and head of the caudate nucleus [5].

MLSA aneurysms have been reported in the literature but are rare. Nomura et al. [6] have reported 62 cumulative cases over 30 years in a literature review. Treatment included 22 surgically treated via clipping, trapping, or resection; 17 out of 20 cases where initial conservative/no interventions were undertaken had spontaneous disappearance or near disappearance. Six underwent endovascular embolization [6]. Vargas et al. [7] have extensively retrospectively reviewed all patients who were diagnosed over a two-year period; 27 were surgically treated via clipping or vessel sacrifice, five endovascular with onyx, or n-butyl-2-cyanoacrylate, and 13 had conservative/no treatment.

The only similar case reported in the literature using a minimally invasive operative technique was reported by Saito et al. [3]. Initially, their patient presented to the hospital without an MLSA aneurysm after initial imaging, but on day 22, the aneurysm was visualized. At that time, the transsulcal-transventricular approach using a tubular retractor was utilized to minimize the exposure and deficit to surrounding tissue due to the difficult visualization with an endoscopic approach [3]. 

Our case adds to the current literature regarding the management of MLSA aneurysms by describing a successful minimally invasive operative technique to treat these aneurysms. Although conservative management has been used with aneurysm resorption/disappearance, surgical or endovascular treatment represents the prominent method of management [3]. Under certain circumstances or clinical conditions such as advanced age of the patient, multiple medical comorbidities, or poor neurologic status, conservative management may be appropriate. However, in patients of younger age and in the presence of associated AVM, which may make spontaneous disappearances less likely, surgical treatment is appropriate [6]. In our patient, endovascular treatment was initially planned; however, due to the small vessel diameter of the MLSA, open surgical management was ultimately performed.

Our surgical management involved a minimally invasive, image-guided, transcortical-transventricular approach to the lateral ventricle via an endoscope. Image guidance allowed a direct approach to the aneurysm and associated hemorrhage. However, poor visualization via the endoscope, related to technical issues, required conversion to a microscopic approach through a slightly enlarged endoscopic tract. Our operative video demonstrates initially attempted clipping and subsequent successful aneurysm excision (Video 1).

Distal MLSA aneurysm presenting with hemorrhage, in particular with IVH, can be effectively treated with minimally invasive open surgical techniques [8]. This direct approach, although contrary to the usual aneurysm approach where proximal vessel control is achieved, avoids extensive dissection of the basal cisterns, and major parent vessel manipulation within an area of small vulnerable, perforating vessels. With image guidance, a transcortical-transventricular approach provided direct access to the aneurysm and associated hemorrhage, thereby allowing the option for hemorrhage evaluation and aneurysm obliteration. Given the small caliber of the MLSA, any hemorrhage with appropriate microsurgical techniques can be controlled with minimal adverse effects and excellent clinical outcomes.

## Conclusions

We presented a case of a 48-year-old female with a past medical history of hypertension and a four-day history of headaches, abulia, and increasing lethargy who was found to have a left caudate ICH with left lateral ventricle IVH. Diagnostic cerebral angiogram demonstrated a distal left MLSA aneurysm and remote left parietal AVM. Surgical management was performed using a minimally invasive, image-guided, transcortical-transventricular approach with successful excision of the distal left MLSA aneurysm. Our case report demonstrates a novel surgical technique for these rare distal MLSA aneurysms and highlights the safety and efficacy of such an approach.
